# Is life satisfaction higher for citizens engaged in political participation: Analysis based on the Chinese social survey

**DOI:** 10.1371/journal.pone.0279436

**Published:** 2022-12-30

**Authors:** Shaocheng Shi, Zixian Zhang, Tianyi Yang, Jiangyin Wang, Tianyang Li, Jinxu Zhao, Tianlan Liu, Kun Wang, Mingyu Yang, Li He

**Affiliations:** 1 Huazhong Agricultural University (School of Marxism), Wuhan, Hubei, China; 2 Zhongnan University of Economics and Law (School of Philosophy), Wuhan, Hubei, China; 3 Nanjing University of Information Science and Technology (School of Law and Public Affairs), Nanjing, Jiangsu, China; University of Naples L’Orientale, ITALY

## Abstract

**Background:**

Political participation is an important component of civil rights. Several studies have shown that citizens’ political participation not only influences the allocation of public resources, but also has a positive correlation with participants’ life satisfaction. Recently, political participation has become increasingly frequent in China; however, the research on Chinese citizens’ political participation and life satisfaction is insufficient. Therefore, this study examined the relationship between political participation and life satisfaction in the Chinese cultural context, and how this relationship varied under different conditions.

**Methods:**

Based on 8,475 respondents from the 2015 Chinese Social Survey, ordinary least squares modeling was used to investigate the relationship of Chinese citizens’ political participation and their life satisfaction, and the differences that might exist in this relationship.

**Results:**

Political participation was closely related to life satisfaction. Compared with non-political participants, the life satisfaction of political participants was 0.133 units higher, which was significant at the 1% level. Regarding the types of political participation, citizens engaged in institutionalized political participation had higher life satisfaction, whereas citizens engaged in non-institutionalized political participation had lower life satisfaction. Furthermore, two social capitals, namely social tolerance and social trust, were the mediating variables linking political participation to citizens’ life satisfaction.

**Conclusions:**

In China, citizens engaged in political participation had higher life satisfaction, in contrast, citizens engaged in non-institutionalized political participation had lower life satisfaction.

## 1 Background

Political participation refers to the phenomenon in which political subjects engage in political activities. In their book *No Easy Choice*: *Political Participation in Developing Countries*, Huntington and Nelson [1] defined political participation as “the activities of civilians trying to influence government decision-making” and limited the subject to “civilians.” In the context of the present study, the definition of political participation is similar; however, we focused on ordinary citizens’ grassroots political participation activities that are closely connected with their work and life. Political participation can be further divided into institutionalized and non-institutionalized political participation according to whether it is advocated by the government or not. Institutionalized political participation refers to the behavior of individuals to reflect their political demands through moderate means, mainly including participating in village (neighborhood) committee elections and reporting opinions to government departments. Because these behaviors do not adversely affect the social stability, they are advocated and encouraged by the government. In contrast, non-institutionalized political participation is the behavior of reflecting one’s political needs through radical means, mainly including petition, participating in demonstrations, strikes. Because these behaviors disrupt the social order and increase the cost of governance for the government, they are usually restricted by the government. In particular, it is worth noting that although petition is a behavior of political participation allowed by the institution, we should also consider it as non-institutionalized political participation. This is because in practice, petition is when a citizen goes beyond the local government to seek help from a higher level of government [2]. The frequency of petition is used as a key indicator to assess the social governance performance of local governments. If the frequency of petition in a jurisdiction is high, then the local government is likely to receive a lower performance evaluation. This would have a negative impact on local state functionaries’ salaries, benefits and job promotions. Influenced by this rule, local governments usually try their best to prevent people from petition. Based on the above arguments, we believe that petition is non-institutionalized political participation.

With the awakening and dissemination of modern civic consciousness, citizens are increasingly choosing to engage in political participation. Relevant studies show that political participation often provides citizens with indirect and direct instrumental and emotional returns. Indirect returns mainly refer to promoting people’s utility through political results that are more in line with their preferences [3], that is, promoting the introduction of favorable policies. However, this type of return usually has less impact on citizens and does not match the time and energy they expend. Therefore, in this study, we focused mainly on the direct instrumental and emotional returns of political participation for citizens. Political participation not only enables citizens to obtain certain instrumental returns, such as social returns measured by reputation, which enhances the collective recognition of citizens, but also brings rich emotional returns. Emotional returns could, for example, include the satisfaction of exercising one’s civil rights and expressing personal interests [4]. However, although theoretical analysis has shown that citizens engaged in political participation have higher life satisfaction; it is unclear whether this would remain true at the empirical level. Moreover, further research is required, particularly in China, to determine whether the relationship of political participation and life satisfaction is positive. Accordingly, this study aimed to address this problem.

Existing studies have paid little attention to the relationship of Chinese political participation and citizens’ life satisfaction, with most researchers focusing on the relationship between life satisfaction and social capital on political participation [5, 6]. Thus, this study focused on the relationship between Chinese citizens’ political participation and life satisfaction. As most direct returns produced by political participation can be classified as social capital, we further incorporated social capital theory to examine the mechanisms of the relationship between political participation and life satisfaction. This study was the first of its kind to address current gaps in the literature and aimed to contribute in two ways.

First, we discussed the relationship of political participation and Chinese citizens’ life satisfaction and used the social capital theory to analyze the mediating mechanisms involved. Second, based on the characteristics of political participation in China, we divided political participation into institutionalized and non-institutionalized political participation and explored their relationships with citizens’ life satisfaction respectively. In general, the Chinese government supports institutionalized political participation and opposes non-institutionalized political participation. Thus, the Chinese government’s differential attitudes toward institutionalized and non-institutionalized political participation make it possible for these two types of political participation to have opposite correlated with citizens’ life satisfaction.

Compared with previous research, this study used Chinese samples to verify the adaptability of existing research findings in the Chinese context, employed the ordinary least squares (OLS) model to investigate the relationship of political participation and life satisfaction, examined the mediating mechanism from the perspective of social capital, and further analyze the difference in the relationship between political participation and different groups in terms of life satisfaction. This study used data from the 2015 Chinese Social Survey (CSS), and the specific variables evaluated were political participation and life satisfaction.

### 1.1 Conceptual discrimination and literature review

For a long time, psychology has focused more on negative emotion and psychological state such as depression and anxiety, and less on positive emotion and psychological state such as happiness, joy, satisfaction, and optimism. With the development of psychology, scholars have gradually realized that the study of happiness and joy is not the same as the study of negative emotion. The research on positive psychology, such as optimism, joy, satisfaction, and happiness, is growing. These studies formed the prototype of Positive Psychology [7]. Life satisfaction and subjective well-being are important concepts in this area [8, 9]. It’s generally considered that there is a strong link between life satisfaction and subjective well-being [10–12]. Specifically, on the one hand, both life satisfaction and subjective well-being are related to quality of life. Diener [13] defines subjective well-being as an individual perception of the quality of life. And life satisfaction is an overall evaluation of his or her quality of life based on subjective criteria [14]. On the other hand, life satisfaction is a key indicator of subjective well-being [11]. Subjective well-being is a comprehensive judgment of an overall quality of life based on self-defined criteria, which includes both people’s cognitive evaluation of their life situation (life satisfaction) and their emotional experience (positive and negative affect) [13]. But life satisfaction focuses on measuring an individual’s cognitive evaluation of his or her long-term living environment [13].

Previous research findings have conflicted regarding the relationship between life satisfaction and political participation. First, studies on factors that affect life satisfaction have shown a close relationship between political participation and life satisfaction. For example, Vats [15] found that rural women who actively participated in politics were more satisfied with their lives than those who did not. In addition, empirical studies examining the correlation between political participation and life satisfaction and happiness have focused on mature democracies in developed countries and found that citizens living under regimes that grant them greater political freedom are, on average, happier and more satisfied with life than citizens elsewhere [16–18]. Related studies have also been conducted in China. For example, one study showed that political participation affects happiness and life satisfaction both directly and indirectly through social support [19]. However, other studies have found no substantial correlation between political participation and life satisfaction [20, 21]. Further, although the relationship between life satisfaction and political participation has been widely discussed, its correlation and causality remain uncertain. Moreover, there is insufficient empirical research on the relationship between these variables in China. The inconsistencies in the results of extant research appear to stem from differences in the path of the effect of political participation on life satisfaction.

First, one view holds that political participation is a means of self-development, self-expression, and self-actualization through which individuals can make a positive impact on their own lives [22, 23]. Some scholars posit that political participation has “procedural utility” independent of political decision-making results [24]. Thus, people will receive satisfaction from participating in the political process, even if they do not obtain the results they want. In contrast, other scholars believe that political participation is a way for individuals to realize their own interests, while improvement in life satisfaction will lead to self-satisfaction, thereby hindering political participation [25].

Second, the correlation between political participation and life satisfaction may also be influenced by the type of political activity. More specifically, those who are more satisfied with their lives may engage more in traditional forms of political participation but less in conflict-related activities, such as political protests [26, 27]. However, the relationship between different types of political activities and life satisfaction varies. Temkin and Flores-Ivich [28] showed that traditional political participation activities are positively correlated with life satisfaction, such as joining political parties [29]. However, other studies have found that neither traditional political engagement, such as voting, nor less intense political engagement, such as signing petitions or wearing campaign stickers, have correlation with life satisfaction [29, 30].

Finally, there is disagreement regarding the directionality of the causal arrow between life satisfaction and political participation. Specifically, the question remains that it’s both possible that people engaged in political participation have higher life satisfaction and that people are more willing to engage in frequent political participation because they have higher life satisfaction. Proponents of the latter generally believe that people with higher life satisfaction have a stronger tendency to engage in political participation. For example, Zhong and Chen [31] found that Chinese farmers with higher life satisfaction were also more likely to vote in local village elections. Similarly, Flavin and Keane [32] examined the relationship between life satisfaction and political participation using personal data from the 2000 American National Election Study and found that respondents who were more satisfied with their lives were more likely to vote and participate in politics in other traditional ways. Compared with people with lower life satisfaction, those with higher life satisfaction appear to be more rational and fit for democratic decision-making and tend to pay more attention to social and political issues [25, 32]. For those with lower life satisfaction, a lower happiness level will inhibit their motivation and interest in political participation [33]; however, dissatisfaction with life can also promote their willingness to participate in protests [34].

### 1.2 Theoretical basis and research hypothesis

In political participation research, a link between political participation and social capital theory has been long established. The social capital theory is widely used in economics, sociology, political science, and other disciplines. Its concept and connotation vary with different application fields and show multidimensional characteristics.

Hanifan [35] was the first to independently use the concept of social capital as a resource beneficial to individual and community development. Following the direction of Hanifan, Bourdieu [36] systematically interpreted the modern meaning of social capital and proposed that social capital is the aggregate of actual or potential resources, which are connected with the institutionalized social relationship network that is familiar or recognized by all. Although there are many different views on the definition of social capital, most scholars believe that social capital primarily refers to the resources obtained in social networks [37–41]. For example, Leung [42] posited that social capital refers to personal resources accumulated through interpersonal activities that help develop strong social networks and connections between individuals and their communities. Putnam [43] proposed that, at the collective level, social capital is used to describe the norms, trust, network, and vision of an organization, which can promote cooperation among members and thus have higher satisfaction. In short, although there are various understandings of social capital, most extant research defines social capital from the individual and social dimensions, thereby positioning social capital as the social resource individuals own or the resources the collective owns to support its members [44]. Specifically, social capital is a resource that includes a network of relationships, reputation, and support at the individual level and reciprocity, interaction, and a shared vision at the collective level.

Helliwell and Putnam [45] found a connection between social relationships and subjective happiness, while Gundelach and Kreiner [46] posited that social capital was the most important predictor of subjective happiness and that the two were highly correlated. Given that both life satisfaction and subjective happiness are related to quality of life and that life satisfaction is an important dimension of subjective happiness, it is also appropriate to apply social capital theory to the study of political participation and life satisfaction.

Political participation improves social capital, and there is a significant positive correlation with social capital and life satisfaction [47]. Portes [48] defined social capital as participation in organizations and general trust in others. According to this definition, citizens have a certain amount of social capital at the beginning of political participation. Through political participation, their social capital can be increased, people with more social capital have higher life satisfaction [49]. Thus, political participation is not only directly linked to citizens’ life satisfaction by affecting their social capital, but also indirectly through collective social capital. Political participation enhances citizens’ sense of rewards and gains by expanding civil society networks and enhancing their personal social capital, thus having higher life satisfaction. Further, political participation also enables citizens to share common goals and forms an informal norm among them, namely, shared values, beliefs, and vision [50], which improves collective social capital and makes them have higher life satisfaction. Based on the above discussion, the following hypothesis was proposed:

**Hypothesis 1**: There is a positive relationship between political participation and citizens’ life satisfaction.

The strength of the relationship between political participation and citizens’ life satisfaction is determined by the means and channels of political participation. Institutionalized political participation is carried out through moderate means and channels, such citizens have higher life satisfaction, while non-institutionalized political participation does the contrary. On the one hand, most of the citizens’ political participation has a certain purpose. Citizens who participate in institutionalized politics tend to realize their self-worth, and their sense of self-worth is significantly positively correlated with life satisfaction [51]. However, for the citizens who participate in non-institutionalized politics, most of their demands cannot be solved for a long time, and there is a negative relationship between accumulated dissatisfaction and life satisfaction. On the other hand, citizens often get certain rewards for their political participation. Institutionalized political participation is often accompanied by extensive contact and communication with others, which helps citizens to expand their social network and obtain more social capital. The social capital index is positively correlated with life satisfaction [52]. However, irregular non-institutionalized political participation may destroy the existing social capital of citizens and thus lead to lower life satisfaction.

**Hypothesis 2**: There is a significant positive relationship between institutionalized political participation and citizens’ life satisfaction, whereas non-institutionalized political participation has the opposite effect.

Through increasing collective social capital and individual social capital, citizens engaged in political participation have higher satisfaction. On the one hand, broadening civil political participation increases social diversity, which has a positive impact on tolerance [53]. Societies that are more tolerant tend to be happier because they create a more relaxed environment conducive to happiness [54], which results in higher life satisfaction. On the other hand, political participation increases interpersonal communication and has a positive effect on promoting interpersonal trust. The relationship and trust with neighbors have a positive independent influence on citizens’ life satisfaction [45].

**Hypothesis 3**: The variables that mediate the positive relationship between political participation and life satisfaction are the degree of social tolerance and trust of neighbors.

## 2 Methods

Ethics approval for the study was granted by the Research Ethics Committee of the Institute of Sociology at the Chinese Academy of Social Sciences, and all participants provided signed informed consent at the time of participation. The study methodology was carried out in accordance with approved guidelines. Regarding data use, the data were processed anonymously.

### 2.1 Data

The data used in this study were derived from the 2015 CSS, which was organized and implemented by the Institute of Sociology of the Chinese Academy of Social Sciences. The survey used China’s fifth and sixth population censuses as the sampling frame and adopted a probability proportional to the size of the sampling method to collect data on the Chinese public in terms of labor and employment, family and social life, and social attitudes. To ensure the rigorousness of the survey, CSS offered a 3–5 days training course and a variety of interview simulation training sessions for supervisors and interviewers. Moreover, in the quality control section, a certain percentage of questionnaires were reviewed at each survey site, at the provincial and national levels, to ensure the quality of the questionnaires, and all questionnaires were double-entered. Thus, the CSS offers a good representation of China and has been widely used in research on China’s issues. The 2015 CSS covered 31 provinces nationwide, including 151 districts, cities, counties, and 604 villages/neighborhood committees for a total of 10,243 samples. After removing the samples with missing values, 8,476 effective samples were included in the analysis.

### 2.2 Measures

#### 2.2.1 Dependent variable

The dependent variable in this study was life satisfaction. This concept is understood as an individual’s assessment of their overall quality of life [55]. Two ways of measuring this concept exist: one based on a single question [56, 57] and the other based on multiple questions [58]. Most studies have followed the first measure, such as Easterlin’s study on the relationship between economic growth and happiness in China [59]. Cheung and Lucas [60] also found that a single-question measure of life satisfaction did not differ substantially from a multiple-question measure of life satisfaction. Therefore, a single-question approach was used in this study. Specifically, according to Yang et al. [61], this variable was obtained from the following question on the CSS: “In general, what is your level of satisfaction with life?” A life satisfaction score ranging from 1 to 10 was assigned based on respondents’ answers. Higher scores indicated greater life satisfaction.

#### 2.2.2 Independent variable

The independent variable in this study was political participation. The CSS paid particular attention to interviewees’ political participation status. The evaluated political participation activities included the following seven items: “discuss political issues with others,” “report social issues to newspapers, radio stations, and other media,” “report opinions to government departments,” “participate in volunteer activities organized by the government/danwei/school,” “participate in village (neighborhood) committee elections,” “petition,” and “participate in demonstrations, strikes.” The first three of these items indicate that the respondent is openly expressing their political demands on specific issues to achieve his or her interests. The fourth item indicates that the respondent participates in voluntary activities organized by various public sectors. Among them, danwei is a special organizational form commonly adopted by various social organizations in China, and is the foundation of China’s political, economic, and social systems. The typical danwei organization includes not only party and governmental agencies and public institutions but also state-owned enterprises, factories, and collectives. The fifth item is the respondents’ exercise of their right to vote for the head of their own grassroots self-government organization. The sixth item is that the respondents crossed over to higher levels of government to defend their rights after being treated unfairly by the grassroots government. Finally, the seventh item belongs to the more intense form of political participation by breaking the existing order for political expression. In this study, individuals who had never participated in any of the above activities were considered to be non-political participants and assigned a value of 0. Individuals who had participated in one or more activities were considered political participants and assigned a value of 1.

Considering that there are multiple types of political participation and that different types of political participation may be correlated with different levels of life satisfaction, referring to the study by Chi [62], this study further divided political participation into institutionalized political participation and non-institutionalized political participation. Institutionalized political participation was often supported and protected by government departments. Non-institutionalized political participation, by contrast, might place participants in opposition to the government, making it difficult to obtain appropriate protection. These two types of political participation might have very different relationships with life satisfaction. In light of this, we treated “discuss political issues with others,” “report social issues to newspapers, radio stations, and other media,” “report opinions to government departments,” “participate in volunteer activities organized by the government/danwei/school,” and “participate in village (neighborhood) committee elections” as institutionalized political participation while treated “petition,” and “participate in demonstrations, strikes” as non-institutionalized political participation.

#### 2.2.3 Control variables

Focusing on factors related to life satisfaction, and according to Bialowolski and Weziak-Bialowolska’s study [63], we selected the following control variables: gender, age, education level, and marital status as demographic variables and religious beliefs, ethnicity, and status of membership in the Communist Party of China (CPC) as characteristic variables of social attributes. Income was regarded as a characteristic variable of economic status. Hukou and province were regarded as characteristic variables of region. Among them, the hukou system is one fundamental social and political-institutional arrangement in China. People can have either native urban or rural hukou by birth, depending on their parents’ hukou status. Over a long period of institutional evolution and social development, the hukou has gradually become correlated with the benefits available to individuals [64, 65]. Specifically, people with urban hukou can freely dispose of their properties and enjoy the quality educational resources tied to their hukou in the city. And people with rural hukou can enjoy the benefits of village collectives (collective dividends, contracted land) and the benefits that come from tilted policies and resources. Thus, in China, each individual receives different benefits for having different hukou, and these differences in benefits in turn have different effects on the lives of individuals. Based on this, it was necessary to include hukou as a control variable.

#### 2.2.4 Mediating variables

Referring to a previous study [66], we mainly considered two types of social capital, social tolerance and social trust, to measure the relationship between political participation and life satisfaction. Social tolerance was obtained from the respondents’ rating of their degree of social tolerance. The scores ranged from 1–10, with 1 representing very intolerant and 10 representing very tolerant. Social trust was obtained from respondents’ responses on the level of trust in their neighbors, with 1 = “not trusting at all,” 2 = “not very trusting,” 3 = “relatively trusting,” and 4 = “very trusting.”

### 2.3 Model selection

#### 2.3.1 OLS model

To explore the relationship between political participation and life satisfaction, this study first used OLS modeling to conduct a preliminary estimate. The model was set as follows:

$$satisfaction_{i}=\alpha_{0}+\alpha_{1}politics_{i}+\sum \gamma_{m}X_{mi}+\varepsilon_{i}$$ (1)

where *satisfaction*_*i*_ indicates the life satisfaction of the *i*th respondent, *politics*_*i*_ indicates whether the *i*th respondent has ever participated in political behavior, *X*_*mi*_ represents other control variables, *ε*_*i*_ represents the random error term, and *α*_1_ is the coefficient to be estimated, reflecting the extent and direction of the correlation between political participation and life satisfaction.

#### 2.3.2 Mediation effect model

Referring to an existing study [67], this study constructed Models (2) and (3) based on Model (1) to test for potential mediating effects.

$$mediating_{i}=\alpha_{0}+\alpha_{2}politics_{i}+\sum \gamma_{m}X_{mi}+\varepsilon_{i}$$ (2)

$$satisfaction_{i}=\alpha_{0}+\alpha_{3}politics_{i}+\sum \beta_{2k}Mediating_{ki}+\sum \gamma_{m}X_{mi}+\varepsilon_{i}$$ (3)

where *mediating*_*i*_ represents the mediating variable. Based on the previous analysis, social tolerance and social trust were selected as mediating variables in this study. The other variables were consistent with the explanation of Model (1).

According to a study by Baron and Kenny [67], there were three steps to test for mediating effects. The first step was to test model (1), and if *α*_1_ was significant, then go to the second step, otherwise, the mediating effect does not exist. The second step was to test models (2) and 3). If both *α*_2_ and *β*_2*k*_ were significant, there is a mediating effect. If at least one of them was insignificant, the third step of the Sobel test was required. Passing the Sobel test implies that there is a mediating effect; failing to pass indicated there was no mediating effect. The formula for the Sobel test is as follows:

$$\text{z}=\frac{\alpha_{2}\beta_{2}k}{\sqrt{\alpha_{2}^{2}S_{\beta }^{2}+\beta_{2k}^{2}S_{\alpha }^{2}}}$$ (4)

where *S*_*α*_ and *S*_*β*_ are the standard deviations of the estimates of the parameters *α*_2_ and *β*_2*k*_, respectively.

#### 2.3.3 PSM model

It is difficult to overcome the endogenous problem of the reverse causality between political participation and life satisfaction using traditional methods; therefore, this study used the propensity score matching method (PSM) proposed by Rosenbaum and Rubin [68] to test the robustness of the relationship between political participation and life satisfaction. The specific steps were as follows:

The first step involved calculating the propensity score using the logit regression model:

PS(X)=Pr{D=1|X}=E{D|X} (5)

where D is a dummy variable of whether an individual engages in political participation. If the answer was yes, then D = 1; otherwise, D = 0. X represents the covariates that affect whether an individual will engage in political participation.

In the second step, the nearest neighbor matching, kernel matching, and radius matching methods were used to match the treatment and control groups based on propensity scores and to calculate the life satisfaction effect of political participation, that is, the average treatment effect (ATT).

The k-nearest neighbor method matches a political participant with the k nearest non-political participants in terms of propensity score and then estimates the effect of political participation on life satisfaction by comparing a given political participant’s life satisfaction with the mean value of life satisfaction of the matched non-political participants. The k-nearest neighbor estimator of the impact of political participation (*ATT*^*k*-*nearst*^) reads:

$$ATT^{k-nearst}=\frac{1}{n}\Sigma_{i=1}^{n}(y_{1i}-\frac{1}{k}\Sigma_{j=1}^{k}y_{oj})$$ (6)

where *n* represents the number of political participants, *y*_1*i*_ represents the life satisfaction of political participant I, and *y*_*0j*_ represents the life satisfaction of non-political participant j.

Kernel matching is a non-parametric method. It estimates the relationship between political participation and life satisfaction by comparing each political participant with a weighted average of life satisfaction of all the non-political participants. The kernel estimator of the impact of political participation (*ATT*^*kernal*^) reads:

$$ATT^{kernel}=\frac{1}{n}\Sigma_{i=1}^{n}(y_{1i}-\Sigma_{l=1}^{n}w_{il}y_{ol})$$ (7)

*w*_*il*_ is defined as:

$$w_{il}=G\left(\frac{p_{1i}-p_{ol}}{b}\right)/\sum_{l=1}^{n}G\left(\frac{p_{1i}-p_{ol}}{b}\right)$$ (8)

where *p*_1*i*_ is the propensity score for political participant i and *p*_*ol*_ is the propensity score for non-political participant l. G(.) denotes the Gaussian kernel function and b denotes a pre-defined bandwidth.

Radius matching matches a political participant with all non-political participants within a predefined propensity score range. The radius estimator of the impact of political participation (*ATT*^*radius*^) reads:

$$ATT^{radius}=\frac{1}{n}\Sigma_{i=1}^{n}(y_{1i}-\frac{1}{r}\Sigma_{m=1}^{r}y_{om})$$ (9)

where *r* denotes the number of non-political participants within the predefined propensity score range and *y*_*om*_ denotes the life satisfaction of non-political participant *m*.

#### 2.3.4 Instrumental variable model

Considering the possible reverse causality between political participation and life satisfaction, we further used the instrumental variable method to address the endogeneity problem. The selection of instrumental variables should meet two basic conditions: first, instrumental variables are highly correlated with endogenous explanatory variables; second, instrumental variables have no direct impact on explained variables. Based on the prerequisites of the instrumental variables, we chose monthly transportation spending and local identity as the instrumental variables of political participation. In American political practice, the dominant resource mobilization theory holds that political participation requires time, money, and political skills as necessary resources [69]. This applies not only to American political practice, but also to other countries. In political practice around the world, time and money are important resources in the process of political participation. They can influence the willingness and actions of individual political participation. However, in extending this theory to China, some scholars argue that political connection is a more important political resource than time, money. Because it can help overcome the political fear and uncertainty faced by political participants in authoritarian regimes [70]. Based on the above theoretical perspectives, we selected respondents’ monthly transportation spending and local identity as instrumental variables for political participation. On the one hand, monthly transportation spending is highly correlated with respondents’ time and money, and respondents who are locals are more likely to have political connection. That is, both variables influence the possibility for individuals to engage in political participation. On the other hand, there is insufficient evidence that monthly transportation spending or local identity directly affects individual life satisfaction. Therefore, these two variables met the requirements of instrumental variables.

## 3 Results

### 3.1 Descriptive statistics

Table 1 shows the descriptive statistics of the sample data. Life satisfaction in the sample population was relatively high, with a mean score of 6.423. Political participants accounted for 62% of the total, reflecting the extensive political participation of Chinese citizens. In addition, of the total sample respondents, 5213 individuals (61.5%) engaged in institutionalized political participation behaviors, whereas 340 individuals (4%) engaged in non-institutionalized political participation behaviors. In terms of mediating variables, the mean score of the sample’s evaluation of social tolerance was 6.168, whereas the mean value of the evaluation of social tolerance was 6.168, which was neither high nor low. In contrast, social trust was high, at 3.019. Regarding the control variables, 48.9% of the sample included men, and the mean age of 47.7 years. The vast majority of the sample was married. Furthermore, 36.3% of the sample had an education level of elementary school or below, whereas 13.8% of the sample had an education level of university or above. Regarding region distribution, 32.6% of the sample resided in urban households, whereas 37.4% resided in eastern provinces.

**Table 1:** Variable definition and descriptive statistics.

Variable	Variable definitions	Obs.	Mean	SD	Min	Max
Life satisfaction	Assign a value from 1−10; the higher the score, the more satisfied with life	8475	6.423	1.94	1	10
Political participation	Yes = 1, No = 0	8475	0.62	0.485	0	1
Institutionalized political participation	Yes = 1, No = 0	8475	0.615	0.487	0	1
Non-institutionalized political participation	Yes = 1, No = 0	8475	0.04	0.196	0	1
Gender	Male = 1, Female = 0	8475	0.489	0.5	0	1
Age	Actual age of the respondents	8475	47.661	13.169	18	70
Age2	Age*Age/100	8475	24.45	12.195	3.24	49
Marital status						
Single	Single = 1, Other = 0	8475	0.078	0.268	0	1
Married	Married = 1, Other = 0	8475	0.858	0.349	0	1
Divorced	Divorced = 1, Other = 0	8475	0.02	0.14	0	1
Widowed	Widowed = 1, Other = 0	8475	0.042	0.2	0	1
Cohabiting	Cohabiting = 1, Other = 0	8475	0.003	.053	0	1
Education level						
Elementary school or below	Elementary school or below = 1, Other = 0	8475	0.373	0.484	0	1
Junior high school	Junior high school = 1, Other = 0	8475	0.323	0.468	0	1
High school	High school = 1, Others = 0	8475	0.166	0.372	0	1
College	College or above = 1, Other = 0	8475	0.138	0.345	0	1
Religious beliefs	Yes = 1, No = 0	8475	0.151	0.358	0	1
Ethnicity	Han ethnicity = 1, Minority ethnicity = 0	8475	0.921	0.27	0	1
CPC	Member of the Communist Party of China = 1, no = 0	8475	0.108	0.31	0	1
Income	Respondent’s personal total income last year, transformed by the logarithm	8475	9.504	1.361	3.401	15.611
Registered residence	Urban = 1, Rural = 0	8475	0.326	0.469	0	1
Province	Eastern province = 1, Other province = 0	8475	0.374	0.484	0	1
Social tolerance	Assign values from 1 to 10, 1 = completely intolerant, 10 = completely tolerant	8439	6.168	1.667	1	10
Social trust	1 = not at all trusting, 2 = not very trusting, 3 = more trusting, 4 = very trusting	8293	3.019	0.621	1	4

CPC: Communist Party of China

### 3.2 Benchmark regression

Table 2 shows the benchmark regression results. Model 1 was the regression of life satisfaction on political participation; Model 2 added a series of control variables based on Model 1, and Models 3 and 4 examined the relationship between different types of political participation and life satisfaction. As seen in Model 2, the coefficient of political participation was positive and rejected the null hypothesis at the 1% level, indicating that after controlling for respondents’ demographic characteristics, social attribute characteristics, economic status characteristics, and region characteristics, there was a significant positive relationship between political participation and life satisfaction. Thus, Hypothesis 1 was supported.

**Table 2:** Benchmark regression results.

	Model 1	Model 2	Model 3	Model 4
	Satisfaction	Satisfaction	Satisfaction	Satisfaction
Political participation	0.191***	0.133***		
	(0.044)	(0.044)		
Institutionalized political participation			0.144***	
			(0.043)	
Non-institutionalized political participation				-0.360***
				(0.118)
Gender		-0.190***	-0.191***	-0.171***
		(0.044)	(0.044)	(0.044)
Age		-0.119***	-0.119***	-0.117***
		(0.012)	(0.012)	(0.012)
Age2		0.136***	0.136***	0.135***
		(0.013)	(0.013)	(0.013)
(Single)				
Married		0.462***	0.462***	0.461***
		(0.104)	(0.104)	(0.104)
Divorced		-0.357*	-0.357*	-0.348*
		(0.195)	(0.195)	(0.195)
Widowed		-0.230	-0.230	-0.246
		(0.153)	(0.153)	(0.153)
Cohabiting		-0.238	-0.237	-0.252
		(0.399)	(0.399)	(0.396)
(Elementary school or below)				
Junior high school		0.129**	0.129**	0.144***
		(0.055)	(0.055)	(0.054)
High school		0.307***	0.306***	0.325***
		(0.068)	(0.068)	(0.067)
College		0.703***	0.700***	0.732***
		(0.085)	(0.085)	(0.084)
Religious beliefs		0.046	0.046	0.055
		(0.061)	(0.061)	(0.061)
Ethnicity		-0.233***	-0.233***	-0.235***
		(0.082)	(0.082)	(0.082)
CPC		0.353***	0.351***	0.367***
		(0.067)	(0.067)	(0.066)
Income		0.201***	0.201***	0.200***
		(0.019)	(0.019)	(0.019)
Registered residence		-0.153***	-0.151***	-0.171***
		(0.053)	(0.053)	(0.052)
Province		0.085**	0.085**	0.077*
		(0.043)	(0.043)	(0.043)
_cons	6.305***	6.469***	6.465***	6.519***
	(0.035)	(0.299)	(0.299)	(0.299)
N	8475	8475	8475	8475
r2	0.002	0.066	0.066	0.066

Note: Robust standard errors are in parentheses; *, **, and *** indicate significance at the 10%, 5%, and 1% levels, respectively.

CPC: Communist Party of China

Models 3 and 4 reported the relationship between different types of political participation and life satisfaction. We found that the coefficient of institutionalized political participation was positive, whereas that of non-institutionalized political participation was negative; both results were significant at the 1% level. Thus, citizens engaged in institutionalized political participation had higher life satisfaction, whereas those engaged in non-institutionalized political participation had lower life satisfaction. Hypothesis 2 was supported.

Regarding the other control variables, men had significantly lower life satisfaction compared to women. The relationship between happiness and age followed a U-shaped curve. Those who were single had lower life satisfaction than those who were married but were higher than those who were divorced. The more educated one was, the more satisfied one felt with life. CPC members had higher life satisfaction than non-CPC members. Increased income came with higher life satisfaction. Residents of eastern provinces had higher life satisfaction compared to residents of non-eastern provinces.

### 3.3 Endogenous analysis

Table 3 reports the results of the two-stage least square regression after using instrumental variables. Column (1) showed the benchmark regression results used for comparison, Column (2) showed the first-stage regression results for the instrumental variables, and Column (3) showed the second-stage regression results for the instrumental variables. As can be seen, both instrumental variables in the first stage were significantly and positively correlated with political participation, indicating that transportation spending and being a local were strongly correlated with the endogenous explanatory variables. The first-stage statistic value of *F* was much larger than the empirical value of 10, which significantly excluded the problem of “weak instrumental variables.” Meanwhile, the Sargan statistic value of 0.543 could not reject the original hypothesis of “all instrumental variables are exogenous,” so the two instrumental variables could be considered as exogenous and not related to the disturbance term. As seen in Column (3), the estimated coefficient of political participation using instrumental variables was significantly positive, further validating the positive correlation between political participation and life satisfaction. However, in quantitative terms, the estimated coefficient of political participation increased in absolute value, suggesting that potential endogeneity led to an underestimation of the enhancing effect of political participation on individual life satisfaction.

**Table 3:** Instrumental variable method: 2SLS.

	(1)	(2)	(3)
	OLS	First-stage	Second-stage
Political participation	0.133***		1.039***
	(0.044)		(0.335)
Monthly transportation spending		0.022***	
		(0.003)	
Local identity		0.140***	
		(0.013)	
F statistic		74.449	
Sargan statistic		0.543	
N	8475	8475	8475

Note: *** indicates significance at the 1% level.

### 3.4 Robustness test

**Fig 1:**
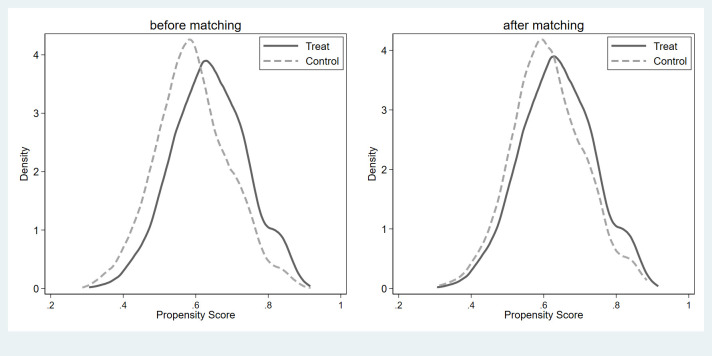
Results of balance hypothesis test: Political participation as a whole.

**Fig 2:**
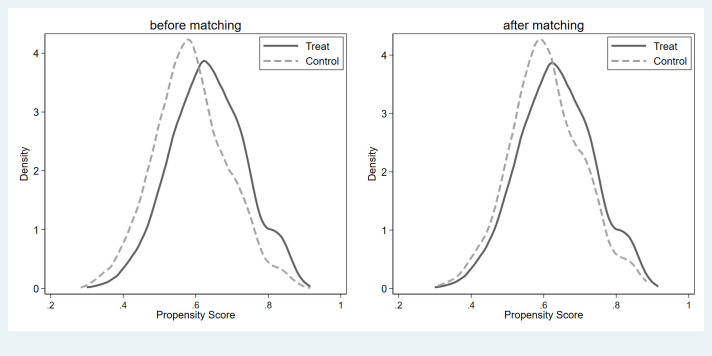
Results of balance hypothesis test: Institutionalized political participation.

**Fig 3:**
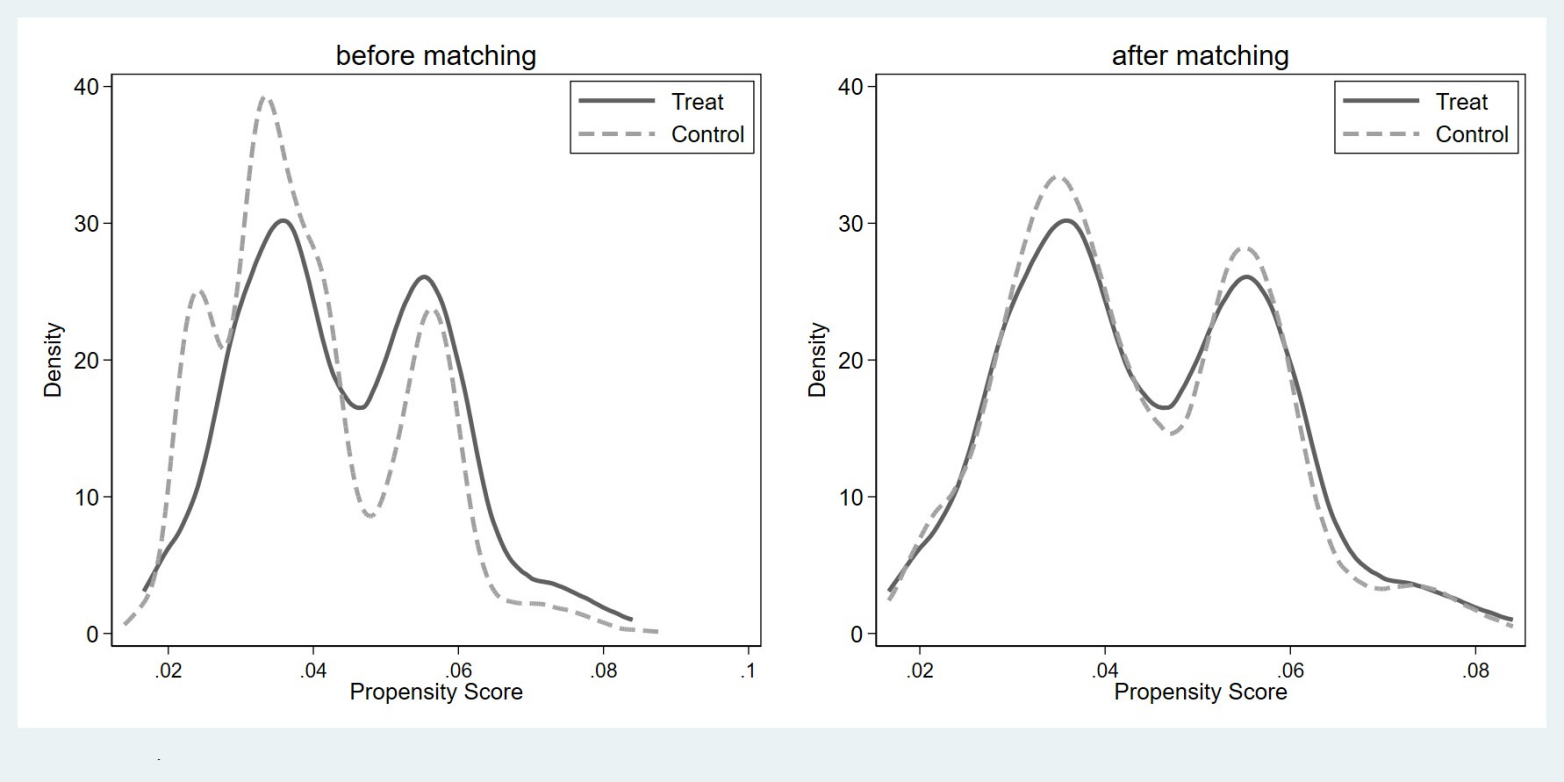
Results of balance hypothesis test: Non-institutionalized political participation.

Changes in life satisfaction might be related to differences in many aspects between political participants and non-political participants, indicating a problem with self-selection. Therefore, if we only used regression analysis to estimate the relationship between political participation and life satisfaction, the estimated results would be biased. Accordingly, this study used PSM to address the endogenous issues. Prior to PSM, it was necessary to perform a balance test, the results of which are shown in Figs 1–3. As can be seen, there was a specific difference and peak deviation between the treatment and control groups before matching. After matching, the specific difference and peak deviation between the treatment and control groups were significantly mitigated, and all covariate deviations were less than 10%, proving that the balance test was passed.

After passing the balance test, different matching methods were used to calculate ATT values in this study: (1) K-nearest neighbor matching where K was set to 1. (2) Kernel matching. This study used the default kernel function and bandwidth. (3) Radius matching. After optimal calculation, the radius was set to 0.03. Table 4 shows that the regression coefficients were consistent with the benchmark regression results under the three matching methods, indicating that the benchmark regression results were robust to a certain extent.

**Table 4:** Average treatment effect.

Variable	Matching methods	ATT	Bootstrap standard error	T-value
Political participation	K-nearest neighbor matching	0.150**	0.058	2.53
	Kernel matching	0.129**	0.043	2.79
	Radius matching	0.151**	0.058	2.54
Institutionalized political participation	K-nearest neighbor matching	0.146**	0.067	2.44
	Kernel matching	0.143***	0.043	3.09
	Radius matching	0.147**	0.064	2.45
Non-institutionalized political participation	K-nearest neighbor matching	-0.355**	0.174	-2.48
	Kernel matching	-0.377***	0.127	-3.11
	Radius matching	-0.364**	0.177	-2.52

Note: **, and *** indicate significance at the 5%, and 1% levels, respectively.

In addition, considering that political attitudes might affect both individual political participation and life satisfaction, we included the political attitudes as a control variable to ensure the robustness of the benchmark regression results. Political attitudes were measured by respondents’ agreement with “government is acting in accordance with the law and enforcing the law fairly.” The values were 1, 2, 3 and 4, indicating “strongly disagree,” “relatively disagree”, “relatively agree” and “strongly agree” respectively. The regression results are shown in Table 5. It can be seen that political attitudes were significantly and positively correlated with life satisfaction, and individuals with positive political attitudes had higher life satisfaction. Political participation and institutionalized political participation remained positively correlated with life satisfaction, while non-institutionalized political participation was significantly negatively correlated with life satisfaction. This was consistent with the benchmark regression results and further demonstrated the robustness of our findings.

**Table 5:** Regression results controlling for political attitudes.

	(1)	(2)	(3)
	satisfaction	satisfaction	satisfaction
Political participation	0.128***		
	(0.043)		
Institutionalized political participation		0.137***	
		(0.043)	
Non-institutionalized political participation			-0.239**
			(0.117)
Political attitudes	0.383***	0.383***	0.379***
	(0.026)	(0.026)	(0.026)
Control Variables	yes	yes	yes
N	8475	8475	8475
r2	0.094	0.094	0.094

Note: Robust standard errors are in parentheses; *, **, and *** indicate significance at the 10%, 5%, and 1% levels, respectively.

### 3.5 Mechanism analysis

The above empirical results suggest that political participation, both institutionalized and non-institutionalized political participation were significantly correlated with citizens’ life satisfaction. This section would continue to explore the mechanisms of the relationship between political participation and life satisfaction. Table 6 reports the results of estimating the effects of the core independent variables on the mediating variables using OLS models. As seen in Models 5–8, the estimated coefficients of political participation and institutionalized political participation on both mediating variables were positive, passing the 1% and 5% significance tests, respectively. This suggests that political participation and institutionalized political participation significantly increased individuals’ perceptions of social tolerance and social trust. Models 9 and 10, however, show that non-institutionalized political participation significantly reduced citizens’ social tolerance and social trust, and the results were significant at the 1% level.

**Table 6:** Results of estimation of independent variables on mediating variables.

	Model 5	Model 6	Model 7	Model 8	Model 9	Model 10
	Social tolerance	Social trust	Social tolerance	Social trust	Social tolerance	Social trust
Political participation	0.091**	0.055***				
	(0.038)	(0.014)				
Institutionalized political participation			0.093**	0.060***		
			(0.038)	(0.014)		
Non-institutionalized political participation					-0.354***	-0.109***
					(0.102)	(0.037)
Control variables	Yes	Yes	Yes	Yes	Yes	Yes
N	8439	8293	8439	8293	8439	8293
r2	0.016	0.057	0.016	0.057	0.017	0.056

Note: Robust standard errors are in parentheses; ** and *** indicate significance at the 5% and 1% levels, respectively.

Table 7 reports the regression results with the inclusion of explanatory and mediating variables. Both social tolerance and social trust were statistically significant after the inclusion of mediating variables, indicating that mediating effects existed on both paths. Specifically, the absolute values of the coefficients of political participation, institutionalized political participation, and non-institutionalized political participation all became smaller after the inclusion of mediating variables, indicating that both political participation and institutionalized political participation were correlated with higher perceived social tolerance and trust of neighbors, and that people with higher perceived social tolerance and trust of neighbors also had higher life satisfaction, while non-institutionalized political participation was the opposite of them.

**Table 7:** Estimation results after adding independent and mediating variables.

	Satisfaction	Satisfaction
Political participation	0.100**	0.114***
	(0.041)	(0.044)
Social tolerance	0.349***	
	(0.014)	
Social trust		0.343***
		(0.037)
Control variables	yes	yes
N	8439	8293
r2	0.155	0.077
Institutionalized political participation	0.110***	0.122***
	(0.041)	(0.044)
Social tolerance	0.349***	
	(0.014)	
Social trust		0.343***
		(0.037)
Control variables	yes	yes
N	8439	8293
r2	0.155	0.077
Non-institutionalized political participation	-0.224*	-0.293**
	(0.116)	(0.118)
Social tolerance	0.348***	
	(0.014)	
Social trust		0.344***
		(0.037)
Control variables	yes	yes
N	8439	8293
r2	0.155	0.077

Note: Robust standard errors are in parentheses; *, **, and *** indicate significance at the 10%, 5%, and 1% levels, respectively.

In addition, we conducted heterogeneity analysis and explored the relationship between political participation intensity and life satisfaction (S1 Appendix).

## 4 Discussion

### 4.1 Political participation and life satisfaction

Political participation is significantly, strongly and positively correlated with life satisfaction [29]. Ordinary citizens who engage in political participation often obtain certain returns or benefits, including social capital. Higher social capital may have positive effects on various aspects of physical and mental health and may improve happiness [71]. Thus, there is a correlation between political participation and life satisfaction. In general, by virtue of the bridging role of social capital, political participation is linked to citizens’ life satisfaction in direct or indirect ways.

On the one hand, citizens who engage in political participation have higher social capital, and this direct approach can make citizens have higher life satisfaction. Most citizens who engage in political participation do not share the same social network. In the process of joint political participation activities, they develop a type of “weak relationship.” This weak relationship can increase citizens’ personal social networks, and such extensive connections provide them with better opportunities to obtain useful resources [72]. Further, more resources can promote citizens’ life satisfaction by providing them with more help. In addition, by communicating and interacting with a wider social network, citizens not only improve their personal abilities but also reveal their personal value; their social influence and self-recognition are also enhanced. Accordingly, they experience a higher level of life satisfaction.

On the other hand, citizens who engage in political participation have more collective social capital, which indirectly leads to higher life satisfaction [73]. When citizens jointly engage in collective activities such as political participation, the trust level between them increases, leading to the formation of shared values and beliefs. This further promotes the improvement of overall social trust and the shaping of a good collective social environment, thereby improving collective social capital. In a social environment with a higher degree of mutual assistance and stronger social trust, citizens also have a stronger sense of belonging. As an additional form of social capital, a sense of belonging is closely related to happiness [74] and can promote life satisfaction. Thus, social interactions and general social trust are the main driving factors of social capital that influence life satisfaction [75, 76]. When political participation benefits citizens in the above aspects, their life satisfaction and happiness are higher.

### 4.2 Types of political participation

Factors such as work, education, and income largely determine the degree of personal political participation [4]. Most citizens with relatively stable jobs and incomes and higher education levels tend to participate in politics through orderly, formal, institutionalized channels set by the government, which can make them have higher life satisfaction. Citizens who participate in politics without institutionalization may be the opposite. These two groups have different purposes for political participation. For citizens who participate in institutionalized politics, political participation usually meets their high-level needs, such as participation in social affairs, the realization of civil rights, and self-worth. Positive self-worth is positively correlated with subjective well-being [77], which may also lead to higher life satisfaction. However, for citizens who choose to participate in politics outside of institutionalization, this choice indicates that their demands cannot be expressed and satisfied through legitimate and formal channels, and the accumulated demands and dissatisfaction over a long period will be significantly and negatively correlated with their life satisfaction.

Furthermore, the rewards of political participation are different for the two groups. Individual social capital is positively correlated with life satisfaction [78]. For citizens who participate in institutionalized politics, political participation means the increase of social interaction and the return of social capital, such as the improvement of personal ability, the expansion of social networks, and so on. This not only helps them in life and with careers but also evokes positive emotions through the balance of effort and rewards, which is positively correlated with life satisfaction [79]. However, for the citizens who participate in non-institutionalized politics, gaining practical benefits and increasing social capital is not possible. Moreover, the intense forms and illegal means of non-institutionalized political participation exert a negative influence on their lives and easily destroy their original social capital, such as the relationship between them and grassroots officials and community workers, which are negatively correlated with their life satisfaction.

### 4.3 Mediating variables of political participation

Rahayu and Harmadi [66] believed that social capital includes “tolerance and trust of neighbors.” That is, the degree of social tolerance and the degree of trust in neighbors can both reflect the social capital status of citizens and precisely correspond to the two dimensions of collective and individual social capital.

Political participation increases the collective social capital of citizens. Through political participation, citizens not only actively express their views on politics and society but also increase society’s openness and diversity. Moreover, it expands social networks, allowing citizens to be exposed to different viewpoints in different networks, which is considered key to forming a tolerant society [80]. Increased social tolerance raises people’s awareness of free choice, leading to higher levels of happiness, and expands people’s range of choices, further enhancing happiness [81] and leading to higher life satisfaction.

Moreover, political participation increases citizens’ individual social capital. When citizens engage in political participation, they inevitably interact with others. It is believed that trust in others can be developed and improved through interaction with different social groups [82]. In other words, political participation enables citizens to have more frequent and in-depth social contact with others, especially their neighbors who live in similar circles, thereby enhancing mutual trust. A high level of trust not only satisfies life needs to achieve life goals but also satisfies psychological needs such as love, belonging, and ultimately leads to higher life satisfaction for them [83].

### 4.4 Returning to the practice of local political participation in China

There has long been a stereotypical understanding of the state of political participation in China, particularly at the grassroots level, where elections are seen as a ritual rather than a right [84]. That is, the government provides few opportunities for citizens to participate in politics [85]. However, a growing body of literature suggests that this does not correspond to reality.

In many Chinese cities, forms of participation such as public hearings and many kinds of consultative and deliberative mechanisms have been incorporated into community autonomy [86]. Research shows that this system of self-governance is generally accepted [87]. At the same time, village-based self-governance systems are also widely established in the vast rural areas of China and have achieved relatively better results. There is evidence that rural residents are more likely to vote in state-controlled elections than urban residents [88]. In addition, with the spread of the Internet, it has become increasingly common for Chinese urban and rural residents, especially younger groups among them, to use emerging media to engage in political participation [89]. In this context, many local innovations in political participation based on the use of new media have emerged. For example, the Wenzhou’s Civil Monitory Organization in Zhejiang Province, is an attempt to monitor the government in Wenzhou by building a bridge between citizens and the government through the local media [90]. Studies have shown that the expansion of political participation and the innovation of methods do have a positive impact on expanding the happiness and public welfare behavior of urban and rural residents [19, 91].

However, it is also important to point out that the government often plays a key role in Chinese political participation practices [92]. Those innovative approaches to political participation that emerge from the bottom-up usually need to be endorsed by the government and implemented from the top-down in order to achieve more widespread results [93]. This also means that the Chinese government prefers to see controlled and orderly political participation. In their view, this is the only way to obtain the desired goal of political participation. Conversely, disorderly political participation can only have destructive effects [94], which is similar to the findings of this study. However, it should not be denied that government-led models of political participation often lack flexibility and adaptability, making it difficult to meet the expanding political participation needs of urban and rural residents. Under such circumstances, residents will turn more to non-institutional channels and thus go beyond the government’s control. In view of this, only providing more space for bottom-up political participation innovation can help solve these problems.

## 5 Conclusions

This study used data from the 2015 CSS to investigate the relationship of Chinese citizens’ political participation and life satisfaction and further verify the applicability of extant research findings to Chinese samples. The empirical results show that political participation in China was positively correlated with life satisfaction. This correlation is limited to institutionalized political participation, while non-institutionalized political participation was negatively correlated with life satisfaction. This has enriched the theoretical and empirical work on the link between political participation and life satisfaction. Specifically, the life satisfaction of political participants was 0.133 units higher than that of non-political participants and the difference was significant at the 1% level. Further analysis of the different types of political participation found that institutionalized political participation was correlated with participants having higher life satisfaction, while non-institutionalized political participation was correlated with participants having lower life satisfaction. In addition, tests of mediating effects suggest that both political participation and institutionalized political participation were correlated with higher tolerance and trust, and higher tolerance and trust were correlated with higher life satisfaction for individuals. In contrast, non-institutionalized political participation was correlated with less of both types of social capital, which in turn was correlated with lower life satisfaction for participants.

This study has the following three limitations: First, this study used cross-sectional data, which were correlation in nature and could not determine causality. Although we believe that political participation will improve individual life satisfaction, some scholars put forward a completely opposite causal path based on American experience, arguing that individuals with high life satisfaction are more likely to engage in political participation [32]. Future studies can collect longitudinal data and conduct cross-lag analyses to determine the direction of causality. Second, we divided political participation into institutionalized political participation and non-institutionalized political participation, and found that there were diametrically opposite correlations between these two forms of participation and individual life satisfaction. However, in China’s political practice, there lacks a clear line between institutionalized political participation and non-institutionalized political participation, and it is difficult for citizens to know when they have crossed the politically acceptable red line [95]. Therefore, we still need to further test whether this conclusion is true. Third, the study was based on a unique Chinese context, which might have some limitations in terms of external validity. Officials have always been interested in making government as “remote and incomprehensible” as possible [96]. This is particularly acute in China, where accountability mechanisms are relatively weak. Thus, the political participation that has emerged in China in recent years has come into people’s lives as a novelty. Conversely, whether the link between political participation and people’s life satisfaction in developed countries where political participation is more diverse and institutionalized is as strong as it is in China needs further discussion.

## Supporting information

S1 Appendix Further analysis. (DOCX)

## List of abbreviations

ATT: average treatment effect on the treatment group
OLS: ordinary least squares
CSS: 2015 Chinese Social Survey
CPC: Communist Party of China
PSM: propensity score matching
