# Supplementary material for: Is life satisfaction higher for citizens engaged in political participation: Analysis based on the Chinese social survey
Source: PLoS One. 2022 Dec 30;17(12):e0279436. doi: 10.1371/journal.pone.0279436 (PMC9803185; doi:10.1371/journal.pone.0279436)
Supplement: S1 Appendix — (DOCX) [file pone.0279436.s001.docx]

## S1 Appendix. Further Analysis

## S1.1 Heterogeneity analysis

The relationship between political participation and life satisfaction might be affected by demographic and socioeconomic factors. Drawing on existing research [1,2], this study further analyzed the differences in the relationship between political participation and individual life satisfaction according to various factors, such as gender and age.

S1 Table shows the heterogeneity analysis results. We can see that there was a significant correlation between women who engage in political participation and life satisfaction, but not for men. In terms of marital status, married people who engage in political participation had higher life satisfaction, whereas there was no significant correlation between political participation of unmarried people and their life satisfaction. In terms of provinces, political participation was positively related to life satisfaction of residents in the eastern and northeastern provinces, but not significantly related to life satisfaction of residents in the central and western provinces. In terms of educational attainment, the relationship between political participation and life satisfaction was stronger for those who had attended college compared with those who had not attended college. Moreover, in terms of age, the correlation between political participation and life satisfaction was stronger for middle-aged people compared with young and old people. The results of institutionalized political participation among different groups remained largely consistent with those of general political participation. Except in the case of provinces, the significant correlation between institutionalized political participation and life satisfaction was seen mainly among residents of the western and northeastern provinces.

**S1 Table. Heterogeneity analysis**

|  | By gender | | By marriage | | By education level | |
| --- | --- | --- | --- | --- | --- | --- |
|  | Male | Female | Married | Unmarried | University or above | Lower than university |
| Political participation | 0.063 | 0.182*** | 0.122*** | 0.106 | 0.213** | 0.108** |
|  | (0.064) | (0.060) | (0.046) | (0.119) | (0.105) | (0.047) |
| Control variables | yes | yes | yes | yes | yes | yes |
| N | 4145 | 4330 | 7180 | 1295 | 1167 | 7308 |
| r2 | 0.083 | 0.058 | 0.054 | 0.152 | 0.042 | 0.054 |
| Institutionalized political participation | 0.080 | 0.188*** | 0.130*** | 0.140 | 0.241** | 0.116** |
|  | (0.064) | (0.060) | (0.046) | (0.119) | (0.104) | (0.047) |
| Control variables | Yes | yes | yes | yes | yes | yes |
| N | 4145 | 4330 | 7180 | 1295 | 1167 | 7308 |
| r2 | 0.083 | 0.058 | 0.054 | 0.152 | 0.043 | 0.054 |
| Non-institutionalized political participation | -0.302** | -0.414** | -0.372*** | -0.289 | -0.595* | -0.319** |
|  | (0.140) | (0.208) | (0.131) | (0.270) | (0.311) | (0.126) |
| Control variables | Yes | yes | yes | yes | yes | yes |
| N | 4145 | 4330 | 7180 | 1295 | 1167 | 7308 |
| r2 | 0.084 | 0.057 | 0.055 | 0.152 | 0.042 | 0.054 |

|  | By age group | | | By province | | | |
| --- | --- | --- | --- | --- | --- | --- | --- |
|  | Youth | Middle-aged | Senior | Eastern | Central | Western | Northeastern |
| Political participation | 0.154* | 0.177*** | -0.082 | 0.146** | 0.029 | -0.018 | 0.264*** |
|  | (0.091) | (0.058) | (0.091) | (0.070) | (0.082) | (0.167) | (0.086) |
| Control variables | yes | yes | yes | yes | yes | yes | yes |
| N | 1703 | 4765 | 2007 | 3171 | 2307 | 708 | 2289 |
| r2 | 0.080 | 0.076 | 0.073 | 0.068 | 0.063 | 0.093 | 0.069 |
| Institutionalized political participation | 0.159* | 0.185*** | 0.151** | 0.034 | -0.020 | 0.292*** | 0.151** |
|  | (0.091) | (0.058) | (0.070) | (0.082) | (0.168) | (0.086) | (0.070) |
| Control variables | yes | yes | yes | yes | yes | yes | yes |
| N | 1703 | 4765 | 3171 | 2307 | 708 | 2289 | 3171 |
| r2 | 0.080 | 0.076 | 0.068 | 0.063 | 0.093 | 0.070 | 0.068 |
| Non-institutionalized political participation | -0.125 | -0.352** | -0.427 | -0.311 | -0.697*** | -0.107 | -0.154 |
|  | (0.207) | (0.159) | (0.278) | (0.214) | (0.204) | (0.441) | (0.212) |
| Control variables | yes | yes | yes | yes | yes | yes | yes |
| N | 1703 | 4765 | 2007 | 3171 | 2307 | 708 | 2289 |
| r2 | 0.079 | 0.075 | 0.074 | 0.068 | 0.069 | 0.093 | 0.065 |

Note: Robust standard errors are in parentheses; *, **, and *** indicate significance at the 10%, 5%, and 1% levels, respectively.

Regarding the relationship between non-institutionalized political participation and people, women who engaged in non-institutionalized political participation were more likely to have lower life satisfaction than men. Further, the negative correlation between non-institutionalized political participation and life satisfaction was mainly found in the married group. Regarding education, for those with higher education, non-institutionalized political participation led to lower life satisfaction. In addition, there was a negative relationship between non-institutionalized political participation and life satisfaction of residents in central provinces, but not with residents in other regional provinces. In terms of age, non-institutionalized political participation was mainly correlated with middle-aged people having lower life satisfaction.

## S1.2 Political participation intensity and life satisfaction

Considering that there might be different correlations between political participation intensity and life satisfaction, we further examined this question. We examined the relationship between institutionalized and non-institutionalized political participation intensity and life satisfaction separately. We recoded life satisfaction into three categories (low, medium, high life satisfaction). Because the data did not satisfy the proportional odds assumption, we used the Generalized Ordered Logit Regression for analysis. Based on the number of individual political participation items, we considered individuals engaged in institutionalized political participation with 1-2 items as slight-single institutionalized political participants, while individuals with more than 2 items as heavy-extensive institutionalized political participants. Individuals engaged in non-institutionalized political participation with 1 item were considered as slight-single non-institutionalized political participants, while individuals with 2 items were considered as heavy-extensive non-institutionalized political participants. In addition, we compared each of the four types of political participants with no political participants. Among them, the slight-single institutionalized and non-institutionalized political participants, and the heavy-extensive institutionalized and non-institutionalized political participants were assigned a value of 1. The non-political participants were assigned a value of 0. S2 Table reports the marginal effects of the Generalized Ordered Logit Regression. The results show that in the group with slight-single political participation, institutionalized political participation helped to increase the probability of individuals having high level of life satisfaction, thereby reducing the probability of individuals having low and medium level of life satisfaction; non-institutionalized participation increased the probability of individuals having low level of life satisfaction, thereby reducing the probability of individuals having high level of life satisfaction. Among the heavy-extensive political participation group, institutionalized political participation helped to increase the probability of individuals having high level of life satisfaction, and thus decreasing the probability of individuals having medium level of life satisfaction; the relationship between non-institutionalized political participation and individuals’ life satisfaction is not significant.

**S2 Table. Marginal effects of the Generalized Ordered Logit Regression**

|  | Slight-single political participation | | | Heavy-extensive political participation | | |
| --- | --- | --- | --- | --- | --- | --- |
|  | satisfaction | satisfaction | satisfaction | satisfaction | satisfaction | satisfaction |
|  | low | medium | high | low | medium | high |
| Institutionalized political participation | -0.010* | -0.030*** | 0.040*** | 0.006 | -0.054** | 0.048*** |
|  | (0.006) | (0.011) | (0.011) | (0.014) | (0.024) | (0.024) |
| Control variables | yes | yes | yes | yes | yes | yes |
| Non-institutionalized political participation | 0.039*** | 0.027 | -0.066** | -0.022 | 0.120 | -0.098 |
|  | (0.012) | (0.027) | (0.028) | (0.065) | (0.116) | (0.108) |
| Control variables | yes | yes | yes | yes | yes | yes |

Note: Robust standard errors are in parentheses; *, **, and *** indicate significance at the 10%, 5%, and 1% levels, respectively.

**References**

1. Vats P. Political participation leading to life satisfaction among rural women. Indian J Health Wellbeing. 2017;12: 1533–1534. Available from: <http://www.i-scholar.in/index.php/ijhw/article/view/165384>.
2. Pirralha A. The link between political participation and life satisfaction: A three wave causal analysis of the German SOEP household Panel. Soc Indic Res. 2018;138: 793–807. doi: [10.1007/s11205-017-1661-x](https://doi.org/10.1007/s11205-017-1661-x).
